# Validation of low-volume plasma dilution protocols for human cytomegalovirus DNA quantification using the cobas CMV assay

**DOI:** 10.1128/spectrum.01106-26

**Published:** 2026-08-11

**Authors:** Gyujin Lim, Jun-Ho Kim, Ho-Joong Joo, Hee-Kyoung Jeon, Mi Ra Ryu, Sun Ae Yun, Areum Shin, Doo Ri Kim, Yae-Jean Kim, Tae Yeul Kim, Hee Jae Huh

**Affiliations:** 1 Department of Laboratory Medicine and Genetics, Samsung Medical Center, Sungkyunkwan University School of Medicine 491523, Seoul, South Korea; 2 Center for Clinical Medicine, Samsung Biomedical Research Institute, Samsung Medical Center 371718, Seoul, South Korea; 3 Department of Pediatrics, Samsung Medical Center, Sungkyunkwan University School of Medicine 371718, Seoul, South Korea; 4 Samsung Advanced Institute for Health Sciences and Technology, Sungkyunkwan University 590525 , Seoul, South Korea; Quest Diagnostics Nichols Institute, Chantilly, Virginia, USA

**Keywords:** cytomegalovirus, CMV DNA quantification, viral load, plasma dilution, cobas 6800

## Abstract

Quantitative nucleic acid amplification tests (QNATs) for human cytomegalovirus (CMV) DNA are widely used for diagnosing and monitoring CMV infection. However, automated CMV-QNATs require relatively large plasma volumes, which may limit testing when specimen volume is limited, particularly in pediatric patients. Here, we evaluated the analytical and clinical performance of two plasma dilution protocols (1:2.5 and 1:5) for the cobas CMV assay to enable CMV DNA quantification using reduced plasma volumes. Analytical sensitivity and linearity were assessed using the World Health Organization International Standard for CMV, and precision was evaluated using pooled plasma. Clinical performance was evaluated using 135 plasma specimens by comparing results from diluted samples with those from the standard protocol. The estimated limits of detection for the 1:2.5 and 1:5 dilution protocols were 28.4 and 195.9 IU/mL, respectively, and the corresponding lower limits of quantitation were 200 and 1,000 IU/mL. Both protocols demonstrated acceptable linearity and excellent precision. Qualitative agreement with the standard protocol was high, with positive percent agreements of 97.9% and 94.7% and negative percent agreements of 95.0% and 100% for the 1:2.5 and 1:5 dilution protocols, respectively. Bland–Altman analysis showed minimal mean differences between diluted and undiluted measurements (−0.03 and −0.08 log_10_ IU/mL). Although both dilution protocols showed agreement with the standard assay, the 1:2.5 dilution protocol demonstrated superior analytical sensitivity and a broader linear range. This protocol enables reliable CMV DNA quantification using as little as 200 μL of plasma and may facilitate CMV testing when plasma volume is limited.

**IMPORTANCE:**

Automated quantitative nucleic acid amplification tests for human cytomegalovirus (CMV) DNA require relatively large plasma volumes, which can limit testing in pediatric patients and other settings with insufficient specimen volume. We evaluated 1:2.5 and 1:5 plasma dilution protocols for the cobas CMV assay using WHO International Standard materials and clinical plasma specimens. Both protocols showed strong agreement with the standard undiluted assay, while the 1:2.5 protocol provided better analytical sensitivity and enabled CMV DNA quantification using approximately 200 μL of plasma. Because no manufacturer-recommended plasma dilution protocol is available for this assay, these findings provide practical performance data that may support laboratories implementing low-volume CMV testing on widely used cobas 6800/8800 systems.

## INTRODUCTION

Human cytomegalovirus (CMV) is a clinically significant viral pathogen that causes significant morbidity in immunocompromised patients. CMV infection represents a major complication in recipients of solid organ transplantation (SOT) or hematopoietic stem cell transplantation (HSCT), where uncontrolled viral replication may lead to severe disease and graft dysfunction (1–3). CMV infection can also occur in children with primary or acquired immunodeficiency and in other critically ill pediatric patients (4).

Quantitative nucleic acid amplification tests (QNATs) for CMV DNA are currently the preferred method for detecting and monitoring CMV infection (5). Measurement of CMV DNA load in blood or plasma provides a rapid and sensitive assessment of viral replication and is widely used for several clinical purposes, including surveillance of CMV infection in transplant recipients, guidance of preemptive antiviral therapy, monitoring of treatment response, and detection of virologic relapse (5).

Several automated QNATs are currently available for CMV DNA quantification, enabling standardized, high-throughput testing with minimal hands-on time. Among them, the cobas CMV assay (Roche Molecular Systems, Branchburg, NJ, USA) is widely implemented in clinical laboratories. However, the cobas CMV assay has been approved by the U.S. Food and Drug Administration (FDA) for use only with plasma specimens, and the minimum required plasma volume for testing is approximately 500 μL (6). Obtaining sufficient blood volume for laboratory testing can therefore be challenging in pediatric patients, particularly in neonates, infants, and critically ill children. Because infants have limited circulating blood volume and are susceptible to iatrogenic anemia caused by repeated phlebotomy, minimizing blood sampling is an important consideration in pediatric clinical practice (7–10). Consequently, the available plasma volume may be insufficient to meet the specimen requirements of automated CMV QNATs when CMV DNA testing is required in pediatric patients.

Dilution of plasma specimens may enable viral load quantification when specimen volume is insufficient for the standard protocol (11, 12). However, dilution may reduce analytical sensitivity and affect quantitative accuracy, and therefore requires validation before clinical implementation. Despite the widespread use of the cobas CMV assay, no manufacturer-recommended dilution protocol has been established, and systematic validation of plasma dilution for low-volume CMV testing remains limited.

In this study, we evaluated the analytical and clinical performance of two plasma dilution protocols (1:2.5 and 1:5) for the cobas CMV assay, which correspond to minimum required plasma volumes of approximately 200 and 100 μL, respectively, using World Health Organization (WHO) International Standard (IS) materials and clinical plasma specimens. The aim of this study was to validate an optimized dilution protocol that enables reliable CMV DNA quantification while reducing the required plasma volume, thereby facilitating CMV testing in pediatric patients with limited specimen availability.

## MATERIALS AND METHODS

### Study design and clinical specimens

**Fig 1:**
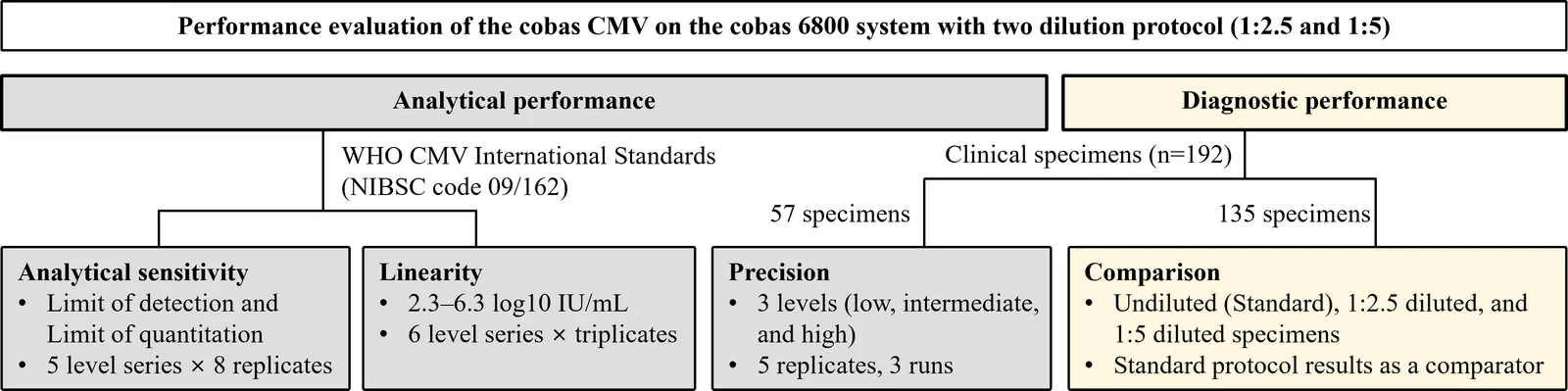
Flow diagram outlining the study design.

We evaluated the analytical and clinical performance of two plasma dilution protocols for the cobas CMV assay performed on the cobas 6800 system (Roche Molecular Systems, Inc.) (Fig. 1). Two dilution protocols (1:2.5 and 1:5) were evaluated to address the limited blood volume that can be obtained from pediatric patients, which may make it difficult to obtain the minimum specimen volume required for testing.

A total of 192 residual clinical plasma specimens originally submitted for CMV DNA quantification were collected from July 2024 to February 2025. Residual specimens remaining after routine testing were stored at –70°C until use in this study. For the clinical performance evaluation, specimens were thawed once and tested using the undiluted standard, 1:2.5 dilution, and 1:5 dilution protocols. Analytical performance was evaluated using the WHO IS for CMV to assess the limit of detection (LOD), lower limit of quantitation (LLOQ), and linearity. For precision analysis, 57 clinical plasma specimens were pooled according to CMV DNA load ranges to generate samples at predefined concentration levels. Clinical performance was evaluated using a total of 135 clinical plasma specimens.

### Specimen preparation and procedures

The WHO CMV IS (NIBSC code 09/162) was used as the CMV reference material for analytical performance evaluation and reconstituted in 1 mL of molecular-grade water, yielding a concentration of 6.70 log_10_ IU/mL. The reconstituted material was then serially diluted with CMV-negative plasma obtained from donors negative for anti-CMV IgG and IgM and confirmed to be negative for CMV DNA using the cobas CMV assay, to prepare target concentrations for analytical evaluation. The clinical plasma specimens for clinical performance evaluation, three pooled clinical specimens prepared for precision analysis, and the prepared WHO IS dilutions were diluted with cobas omni Specimen Diluent (Roche Molecular Systems, Inc., part number 06997511190), a reagent for the cobas 6800/8800 system provided by the manufacturer, at the ratios of 1:2.5 and 1:5, respectively.

The cobas CMV test was performed on the cobas 6800 system, a fully integrated molecular assay system comprising four modules: sample supply, transfer, processing, and analytic modules, according to the manufacturer’s instructions. The standard undiluted protocol requires a minimum plasma volume of approximately 500 μL, with a manufacturer-stated LOD of 20.6 IU/mL and an LLOQ of 34.5 IU/mL. The master mix used in the cobas CMV assay contained a detection probe specific for the CMV DNA polymerase UL54 gene and a probe for DNA-QS, a non-CMV DNA quantitation standard. Every batch included high positive, low positive, and negative controls. The quantified viral loads of diluted samples were multiplied by the dilution correction factor (2.5 or 5) to calculate the actual DNA concentrations.

### Analytical performance

The analytical performance of the cobas CMV assay with the two dilution protocols was evaluated by determining analytical sensitivity (LOD and LLOQ), linearity, and precision. An exploratory analytical sensitivity evaluation was performed using a five-point level series of the WHO CMV IS diluents (Table 1). A total of eight replicates per concentration level were tested for both 1:2.5 and 1:5 dilution protocols. As an exploratory evaluation with a limited number of replicates, the LODs of the two dilution protocols were estimated using probit regression analysis. Confidence intervals for the estimated LODs were not reported because the limited replicate number did not allow sufficiently precise interval estimation. The LLOQs were determined as the lowest nominal concentration equal to or greater than LOD and meeting the criterion that the total error (TE), calculated as |bias| + 2 × standard deviation (SD), was <0.5 log_10_ IU/mL (13). Linearity was evaluated using a six-point level series ranging from 2.3 to 6.3 log_10_ IU/mL prepared from the WHO CMV IS. Three replicates were tested for each concentration. The linearity was assessed by fitting first-, second-, and third-order polynomial regression models within the measured concentration range. For precision evaluation, pooled clinical specimens were used to prepare three concentration levels (low, intermediate, and high) to assess intra- and inter-assay variability. A total of 57 clinical specimens were pooled to generate three target concentration levels: low (2.74 log_10_ IU/mL), intermediate (3.75 log_10_ IU/mL), and high (4.75 log_10_ IU/mL). Intra-assay variability was determined by five replicates within the same run, whereas inter-assay variability was determined using three independent runs. Coefficients of variation (CVs) were calculated to assess the precision.

**TABLE 1:** Exploratory analytical sensitivity evaluation results of the cobas CMV assay for the 1:2.5 and 1:5 dilution protocols^a^

Dilution protocol	Hit rate (no. of positives/replicates) at a CMV DNA load [IU/mL (log_10_ IU/mL)] of						Estimated probit LOD [IU/mL (log_10_ IU/mL)]	LLOQ [IU/mL (log_10_ IU/mL)]
	1000 (3.00)	200 (2.30)	100 (2.00)	50 (1.70)	25 (1.40)	12.5 (1.10)		
1:2.5	ND	8/8	8/8	8/8	7/8	2/8	28.4 (1.45)	200 (2.30)
1:5	8/8	7/8	8/8	6/8	3/8	ND	195.9 (2.29)	1,000 (3.00)

^a^ LLOQ, lower limit of quantitation; LOD, limit of detection; ND, not done.

### Clinical performance

A total of 135 stored clinical specimens were thawed and tested using three methods: (i) undiluted standard protocol; (ii) 1:2.5 dilution protocol, and (iii) 1:5 dilution protocol. Results obtained from the standard protocol using undiluted specimens were used as the comparator method to evaluate the agreement between standard and dilution protocols.

### Data analysis

The agreements between qualitative results obtained using each dilution protocol and the standard protocol were evaluated by calculating positive percent agreement (PPA), negative percent agreement (NPA), and Cohen’s kappa coefficient. The agreements between viral loads measured by each dilution protocol and the standard protocol were analyzed using Bland–Altman plots, and the mean difference and 95% limits of agreement (LOAs) were calculated. The correlation between viral loads was analyzed using Passing–Bablok regression, and Spearman correlation coefficients (*ρ*) were calculated. Statistical analysis was performed using MedCalc Statistical Software version 23.4.9 (MedCalc Software Ltd, Ostend, Belgium) and SPSS version 29 (IBM Corporation, Armonk, NY, USA).

## RESULTS

### Analytical performance

**Fig 2:**
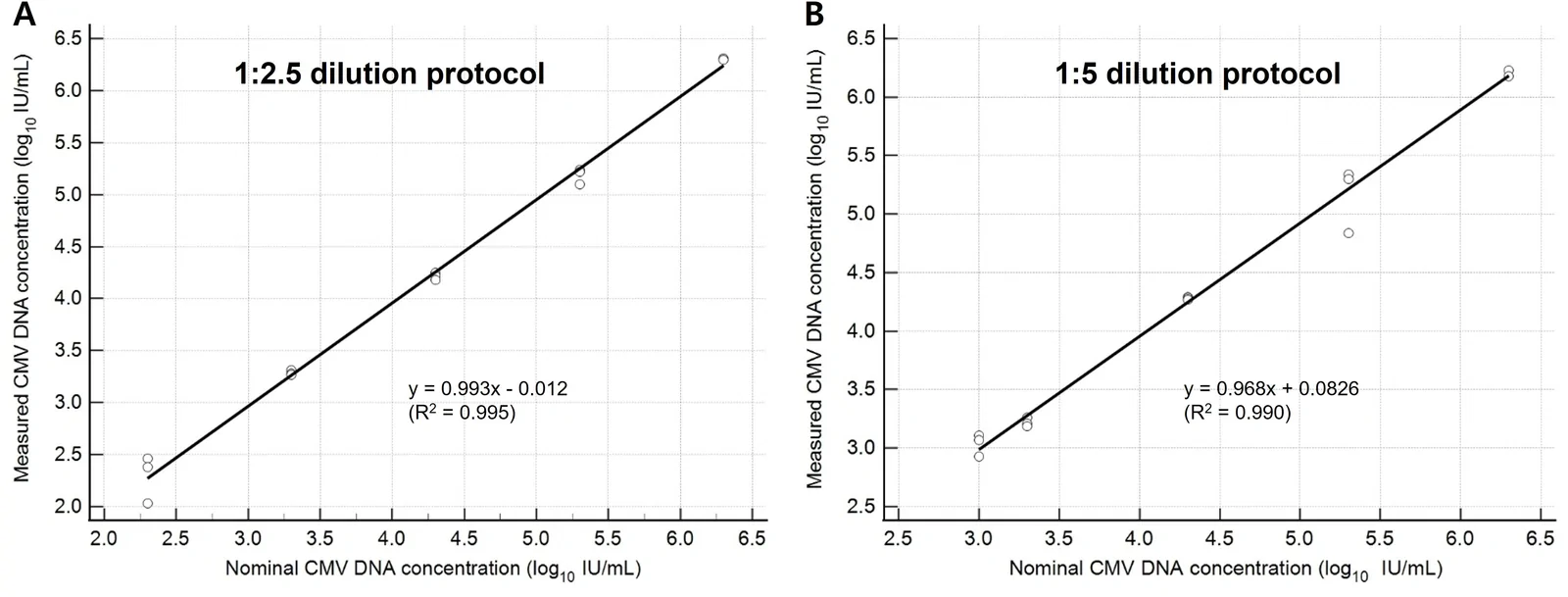
Linear ranges of the cobas CMV assay for the 1:2.5 dilution protocol (**A**) and 1:5 dilution protocol (**B**). The assay showed linearity over a range of 2.3–6.3 log_10_ IU/mL and 3.0–6.3 log_10_ IU/mL for the 1:2.5 and 1:5 dilution protocol, respectively. The solid lines indicate linear regression lines.

The estimated LODs of the cobas CMV assay for the 1:2.5 and 1:5 dilution protocols were 28.39 IU/mL (1.45 log_10_ IU/mL) and 195.9 IU/mL (2.29 log_10_ IU/mL), respectively (Table 1). The LLOQ was 200 IU/mL (2.30 log_10_ IU/mL) for the 1:2.5 dilution protocol and 1,000 IU/mL (3.00 log_10_ IU/mL) for the 1:5 dilution protocol; the calculated TE was 0.34 log_10_ and 0.27 log_10_ IU/mL, respectively. For linearity assessment, the first-order linear regression model provided the best fit in the regression analysis. Linearity was acceptable within the concentration range from the LLOQ to 6.3 log_10_ IU/mL with an R^2^ ≥ 0.990 for both dilution protocols (Fig. 2). The intra- and inter-assay variabilities for both dilution protocols were below 5%, except for the intra-assay variability at the low concentration (2.74 log_10_ IU/mL) for both protocols, which slightly exceeded 5% (Table 2).

**TABLE 2:** Intra- and inter-assay variability of the cobas CMV assay for the 1:2.5 and 1:5 dilution protocols

CMV DNA concentration (log_10_ IU/mL)	1:2.5 dilution protocol		1:5 dilution protocol	
	Intra-assay variability (%CV)	Inter-assay variability (%CV)	Intra-assay variability (%CV)	Inter-assay variability (%CV)
Low (2.74)	5.54	0.62	5.31	0.31
Intermediate (3.75)	1.34	0.28	1.75	0.39
High (4.75)	1.03	0.69	0.81	1.02

### Method comparison

Among the 95 specimens positive by the standard protocol, 90 had quantifiable CMV DNA loads and five were detected below the LLOQ. The median viral load of the 90 quantifiable specimens was 3.64 log_10_ IU/mL (interquartile range, 3.25–3.99 log_10_ IU/mL; range, 1.63–5.16 log_10_ IU/mL). Fifteen specimens (16.7%) had viral loads below 3.0 log_10_ IU/mL, 53 (58.9%) had viral loads of 3.0 to <4.0 log_10_ IU/mL, and 22 (24.4%) had viral loads of ≥4.0 log_10_ IU/mL. When using the results obtained from the standard protocol as a comparator, the agreement between qualitative results obtained with each dilution protocol and the standard method is summarized in Table 3. Both dilution protocols demonstrated almost perfect qualitative agreement with the standard protocol (κ ≥ 0.91). Eight specimens showed discordant qualitative results among the three protocols, all of which occurred at very low CMV DNA levels. Of the six specimens detected by the standard protocol, three had quantifiable viral loads of 1.63, 1.80, and 1.85 log_10_ IU/mL, whereas the remaining three were detected below the LLOQ. The other two specimens were not detected by the standard protocol but were detected below the LLOQ using the 1:2.5 dilution protocol.

**TABLE 3:** Agreement between qualitative results by each dilution protocol and standard protocol^a^

Dilution protocol	Result	Standard protocol		PPA (95% CI)	NPA (95% CI)	Kappa (95% CI)
		Detected	Not detected			
1:2.5 dilution	Detected	93	2	97.9% (92.6%–99.7%)	95.0% (83.1%–99.4%)	0.93 (0.86–0.99)
	Not detected	2	38			
1:5 dilution	Detected	90	0	94.7% (88.1%–98.3%)	100% (91.2%–100%)	0.91 (0.84–0.99)
	Not detected	5	40			

^a^ PPA, positive percent agreement; NPA, negative percent agreement; CI, confidence interval.

**Fig 3:**
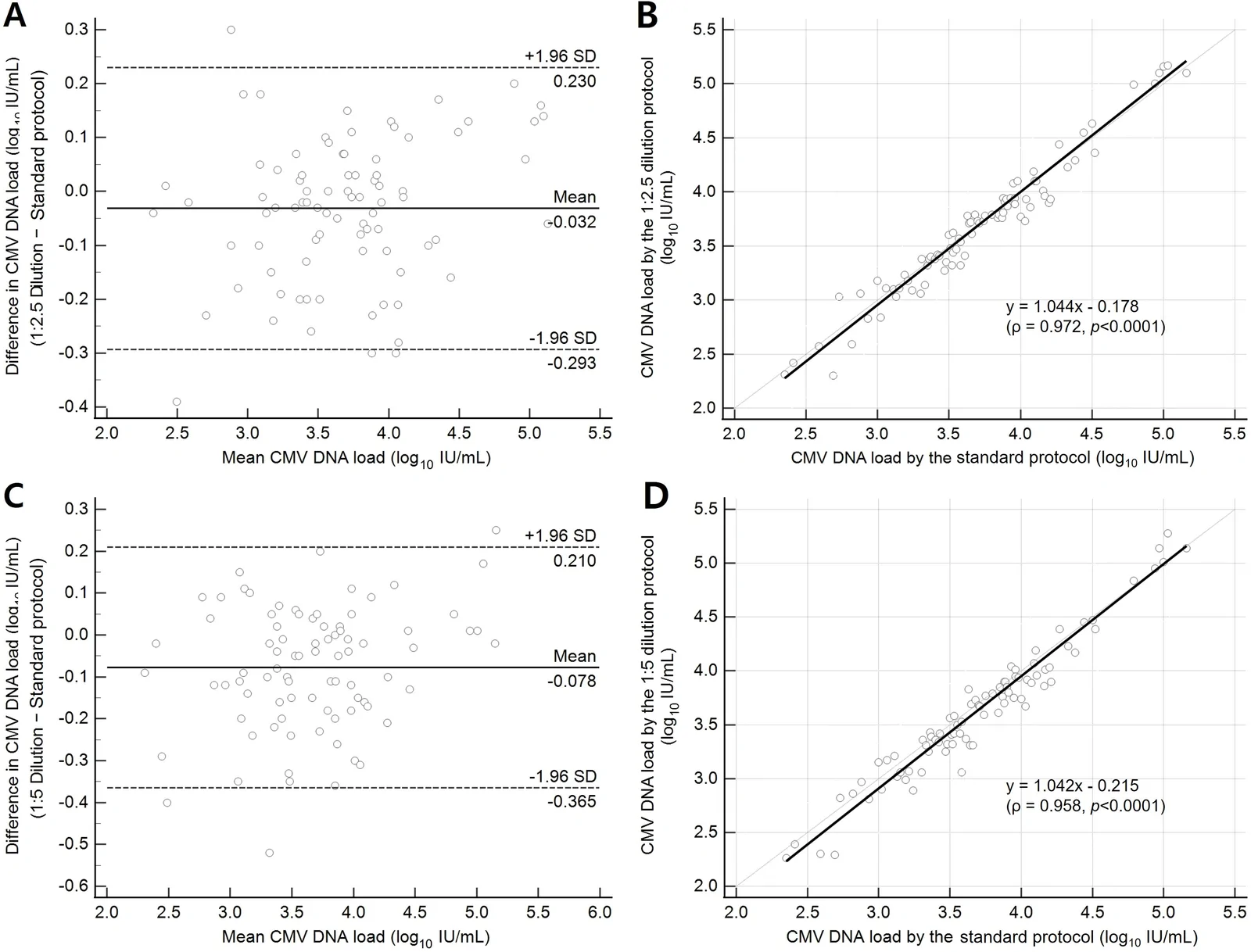
Comparison of CMV DNA loads measured using the 1:2.5 and 1:5 dilution protocols with those measured by the undiluted method. (**A**) Bland–Altman plot and (**B**) Passing–Bablok regression comparing CMV DNA loads measured using the 1:2.5 dilution protocol and the undiluted method. (**C**) Bland–Altman plot and (**D**) Passing–Bablok regression comparing CMV DNA loads measured using the 1:5 dilution protocol and the undiluted method. In the Bland–Altman plots, solid and dashed lines indicate the mean difference and the 95% limits of agreement (mean differences ± 1.96 × standard deviation), respectively.

A total of 84 clinical specimens that were within the linear measurement range for the standard protocol and both dilution protocols were included in Bland–Altman and Passing–Bablok analyses. Bland–Altman analysis showed minimal mean differences between the dilution protocols and the standard protocol. The mean differences were −0.03 log_10_ IU/mL (95% LOA, −0.29 to 0.23 log_10_ IU/mL) for the 1:2.5 dilution protocol (Fig. 3A) and −0.08 log_10_ IU/mL (95% LOA, −0.37 to 0.21 log_10_ IU/mL) for the 1:5 dilution protocol (Fig. 3C), indicating good agreement between the dilution and standard protocols.

Passing–Bablok regression analysis demonstrated a strong correlation between CMV DNA loads measured using the dilution protocols and those measured using the standard protocol (Spearman ρ ≥ 0.99) (Fig. 3B and D). The 95% confidence intervals for the slopes included one and those for the intercepts included 0, indicating no significant proportional or constant bias.

## DISCUSSION

Studies investigating the optimal blood specimen type for CMV DNA quantification have been extensively conducted, particularly comparisons between whole blood and plasma for CMV DNA monitoring (3, 14–16). Although CMV DNA loads measured in whole blood and plasma generally correlate well, viral loads measured in whole blood tend to be higher than those measured in plasma because CMV DNA may also be detected within infected leukocytes (3, 14–16). Nevertheless, several studies have suggested that persistent CMV DNAemia in plasma more accurately reflects active viral replication and may better predict clinically significant CMV infection (2, 15). For this reason, many experts favor plasma over whole blood for monitoring CMV infection, particularly in transplant recipients (2).

Consistent with this preference, the major fully automated high-throughput systems currently used for CMV DNA quantification have all been FDA-approved for use with plasma specimens: these include the cobas CMV assay, the Alinity m CMV AMP kit (Abbott Molecular Inc., Des Plaines, IL, USA), and the Aptima CMV Quant assay (Hologic Inc., San Diego, CA, USA) (6, 17, 18) (Table 4). While these systems provide standardized workflows that enable efficient and reliable CMV DNA quantification in clinical laboratories, they require relatively large plasma volumes for testing. The minimum plasma volumes required range from 500 to 700 μL (6, 17, 18) (Table 4). Moreover, when dead volume is considered, an even larger plasma volume may be required in practice, which consequently increases the amount of blood that must be collected from patients. In clinical laboratories, insufficient specimen volume is therefore occasionally encountered in pediatric patients. Under these circumstances, dilution of plasma specimens may be required when the available specimen volume does not meet the minimum requirement of the assay. The manufacturers of both the Alinity m CMV AMP kit and the Aptima CMV Quant assay provide dilution protocols for plasma specimens, including a 1:2.5 dilution using a dedicated dilution kit for Alinity m and a 1:3 dilution using CMV-negative control for Aptima (17, 18) (Table 4). In contrast, no manufacturer-recommended dilution protocol has been established for the cobas CMV assay. Furthermore, although several studies have evaluated CMV molecular assays using non-plasma specimens such as urine, breast milk, or saliva for the diagnosis of CMV infection (19–21), dilution protocols for plasma specimens have not been systematically investigated. Because the cobas 6800 system is widely implemented in clinical laboratories, the validated dilution protocol proposed in this study may provide a practical laboratory solution when plasma volume is insufficient for the standard assay workflow.

**TABLE 4:** Comparison of specimen requirements and dilution protocols for major automated CMV quantification assays

Assay	Manufacturer	Platform	Approved specimen type	Minimum plasma volume for testing	Dilution protocol provided by manufacturer	Recommended dilution ratio	Recommended diluent
cobas CMV assay	Roche Molecular Systems	cobas 6800/8800	Plasma	500 μL	No	1:2.5 (this study)	cobas omni Specimen Diluent (this study)
Alinity m CMV AMP kit	Abbott Molecular	Alinity m	Plasma	650 μL	Yes	1:2.5	Alinity m Specimen Dilution Kit I
Aptima CMV Quant assay	Hologic	Panther system	Plasma, whole blood	500 µL assay input; 700 µL required in a secondary tube	Yes	1:3	Aptima CMV Negative Control

In this study, we evaluated the analytical and clinical performance of two plasma dilution protocols for the cobas CMV assay and demonstrated that both the 1:2.5 and 1:5 dilution protocols showed strong agreement with the standard undiluted protocol within the dynamic range of the assay. The dilution approaches enabled CMV DNA quantification when the available plasma volume was insufficient for the standard testing protocol, allowing the assay to be performed using as little as 200 μL or even 100 μL of plasma. As expected, the adoption of dilution protocols resulted in reduced analytical sensitivity compared with the standard cobas CMV assay (LOD 20.6 IU/mL and LLOQ 34.5 IU/mL) (6). In particular, the LLOQ increased to 1,000 IU/mL for the 1:5 dilution protocol. Although no universal viral load threshold for CMV management has been established, relatively low levels of CMV DNAemia may still be clinically relevant, particularly in high-risk transplant recipients in whom early initiation of preemptive therapy is recommended (2, 3, 22). Considering this clinical context, the higher LLOQ observed with the 1:5 dilution protocol may limit its sensitivity for detecting low-level DNAemia. In contrast, the 1:2.5 dilution protocol maintained acceptable analytical performance while allowing substantial reduction in required plasma volume, making it more suitable for routine clinical implementation where plasma volume is limited.

However, this study has several limitations. First, the dilution protocols were evaluated using the cobas omni specimen diluent, a reagent supplied by the manufacturer for use with the cobas 6800/8800 system, and alternative diluents, such as phosphate-buffered saline were not assessed. Therefore, the analytical performance of the dilution protocols may differ when other diluents are used. Second, the clinical evaluation was performed using residual plasma specimens obtained from adult patients rather than pediatric patients. Because residual specimens from pediatric patients are often extremely limited in volume, it was not feasible to perform the comprehensive analytical and clinical validation required for this study using pediatric samples. Nevertheless, the use of adult clinical plasma specimens allowed evaluation across a wide range of viral loads and provided practical evidence supporting the analytical reliability of the proposed dilution protocols. Third, the analytical sensitivity evaluation was exploratory and included only eight replicates per concentration level. This number was insufficient for robust estimation of the LOD and its confidence interval and may have contributed to the non-monotonic hit rates observed at adjacent low concentrations. Additional testing with 20 or more replicates per concentration would be required to establish the LOD more precisely. Therefore, the LOD values reported in this study should be interpreted as preliminary estimates.

In conclusion, both dilution protocols demonstrated acceptable clinical performance compared with the undiluted assay; however, the 1:2.5 dilution protocol showed more favorable analytical sensitivity and linear range, making it more suitable for routine clinical implementation. This approach enables reliable CMV DNA quantification with substantially reduced plasma volume and may facilitate CMV testing in pediatric patients with limited specimen availability.

## Data Availability

The de-identified data generated and analyzed during this study are available from the corresponding author upon reasonable request, subject to applicable institutional and ethical requirements.
